# Whole-genome resequencing of Japanese large-sized tomato cultivars provides insights into the history of modern breeding

**DOI:** 10.1270/jsbbs.24004

**Published:** 2024-08-23

**Authors:** Eiji Yamamoto, Hiroshi Matsunaga, Akio Ohyama, Tsukasa Nunome, Hirotaka Yamaguchi, Koji Miyatake, Kenta Shirasawa, Sachiko Isobe

**Affiliations:** 1 Kazusa DNA Research Institute, 2-6-7 Kazusa-Kamatari, Kisarazu, Chiba 292-0818, Japan; 2 Graduate School of Agriculture, Meiji University, 1-1-1 Higashi-Mita, Tama-ku, Kawasaki, Kanagawa 214-8571, Japan; 3 Institute of Crop Science (NICS), National Agriculture and Food Research Organization (NARO), 2-1-2 Kannondai, Tsukuba, Ibaraki 305-8518, Japan; 4 Institute of Vegetable and Floriculture Science (NIVFS), National Agriculture and Food Research Organization (NARO), 360 Kusawa, Ano, Tsu, Mie 514-2392, Japan; 5 Institute of Vegetable and Floriculture Science (NIVFS), National Agriculture and Food Research Organization (NARO), 3-1-1 Kannondai, Tsukuba, Ibaraki 305-8666, Japan

**Keywords:** tomato, whole-genome resequencing, genomic population structure, genomic prediction, genome-wide association study

## Abstract

Tomatoes have the highest agricultural production among vegetables in Japan and worldwide. Japanese large-sized fresh-market tomatoes have a unique breeding history that differs from that of other countries, represented by pink-colored and juicy fruits with a good taste and flavor. We performed whole-genome resequencing of 150 Japanese large-sized fresh-market tomato cultivars released from the 1940s to the 2000s to unveil how breeding selection has changed the genome of Japanese tomato cultivars and provide a genomic basis for future Japanese tomato breeding. The genomic population structure of the cultivars was highly correlated with the year of release. Comparison between the agronomic performance and release year of the cultivars reflected trends in recent breeding selection: an increase in fruit sugar content and a decrease in yield performance. Multiple selection signatures were detected on all the tomato chromosomes. One of the selection signatures was related to the introgression of a resistance gene (*Tm-2*) from a wild relative. Interestingly, some of the putative QTLs detected by genome-wide association studies did not co-localize with the selection signatures, indicating that the genetic diversity of Japanese tomato cultivars still has the potential for genetic improvement of agronomic performance.

## Introduction

Tomato (*Solanum lycopersicum*, 2*n* = 2*x* = 24) is an important vegetable crop with the highest production in the world (Food and Agriculture Organization (FAO) 2023). There are two commercial objectives for tomatoes: fresh-market tomatoes are used for direct consumption, and processed tomatoes are used for processed foods such as ketchup, juices, and soups. In addition, there are two categories based on the average fruit size of the cultivars: cherry tomatoes (small-sized fruit, approximately 10–20 g) and large-sized tomatoes.

Tomatoes also have the highest agricultural production among vegetables in Japan (Ministry of Agriculture, Forestry and Fisheries 2023). Japanese large-sized fresh-market tomatoes have unique breeding histories that differ from those of other countries (Sumita *et al.* 2008, Tomato breeding team, Takii & Company, Limited 2020). Tomato breeding in Japan started in the late 19^th^ century. For fresh-market tomato, Japanese people in those days preferred pink-colored and juicy fruits with high locule numbers. The first F_1_ tomato cultivar (‘Fukuju No. 1’) was developed in 1938, which marked the beginning of modern tomato breeding in Japan. Until the 1970s, high yield, disease resistance, and adaptation to various cultivation conditions were the main breeding objectives for Japanese tomatoes. In the 1980s, good taste and flavor, including high sugar content, became a major breeding objective, and intensive selection of these traits often resulted in a decrease in yield performance. For example, ‘Momotaro’, a representative Japanese tomato cultivar released in 1985, showed +2 degree of Brix (an indicator of fruit sugar content) while its yield performance was 80% of the major cultivars of those days (Higashide *et al.* 2012). Currently, improving both taste/flavor and yield are the most important breeding objectives (Higashide *et al.* 2012).

Nowadays, genomics-assisted breeding plays an important role in efficient crop breeding (Varshney *et al.* 2021). Genomic analyses of a collection of cultivars and/or accessions not only provide insight into the history of domestication and breeding (Lin *et al.* 2014) but also enable various genomics-assisted breeding approaches, including genome-wide association studies (GWAS; Tieman *et al.* 2017) and genomic selection (Yamamoto *et al.* 2016). We performed whole-genome resequencing of 150 Japanese large-sized fresh-market tomato cultivars, including three pure-line and 147 F_1_ cultivars, to unveil how breeding selection changed the genome of Japanese cultivars and provide insights into future Japanese tomato breeding. We then conducted genomic population structure analysis, genomic prediction, GWAS, and selection signature detection.

## Materials and Methods

### Plant materials and whole-genome resequencing

We used a collection of 150 large-sized tomato cultivars that included three pure-line and 147 F_1_ cultivars (Supplemental Table 1). Genomic DNA was extracted from leaves using a DNeasy Plant Mini Kit (Qiagen, Hilden, Germany). The DNA was sheared into approximately 350 bp fragments using Covaris S220 (Covaris, Woburn, MA, USA), and the fragments 300–400 bp in length were fractionated using BluePippin (Sage Science, Beverly, MA, USA). The fractionated DNA was used for DNA library construction using a TruSeq DNA PCR-Free Library Preparation Kit (Illumina, San Diego, CA, USA) in accordance with the manufacturer’s protocols. Sequencing of the DNA library was performed using HiSeq X (Illumina) with 2 × 150 paired-end reads. The reads obtained were subjected to quality control as follows: bases with quality scores less than 10 were filtered using PRINSEQ version 0.20.4 (Schmieder and Edwards 2011), and adaptor sequences in the reads were trimmed using fastx_clipper from FASTX-Toolkit version 0.0.13 (http://hannonlab.cshl.edu/fastx_toolkit/). The filtered reads were mapped onto the S_lycopersicum_chromosomes.3.00.fa (https://solgenomics.net/ftp/tomato_genome/Heinz1706/assembly/build_3.00/) using Bowtie 2 version 2.3.2 (Langmead and Salzberg 2012) with parameters of maximum fragment size length, 1000 (X = 1000), in the ‘—sensitive’ preset of the ‘—end-to-end’ mode. Summary metrics of the read mapping results were obtained using CollectRawWgsMetrics option of Picard version 2.9.0 (https://github.com/broadinstitute/picard/releases/tag/2.9.0). The resulting binary sequence alignment/map format (BAM) files were subjected to variant calling using GATK version 4.1.7.0 with default parameter settings (DePristo *et al.* 2011). The variants were filtered with VCFtools version 0.1.13 (Danecek *et al.* 2011) with parameters of ‘—minDP 5’, ‘—maxmeanDP 40’, and ‘—max-missing 0.9’. Finally, missing genotypes were imputed using BEAGLE version 4.1 (Browning and Browning 2007).

### Linkage disequilibrium (LD)

The variants were filtered with VCFtools version 0.1.13 with ‘--maf 0.05’ to calculate the LD values. Variant pruning based on LD was performed using BCFtools +prune version 0.1.13 (Danecek *et al.* 2011) with options ‘-m 0.99’ and ‘-w 1000’. LD values were calculated using VCFtools version 0.1.13 with ‘--ld-window-bp 3000000’. Options ‘--geno-r2’ and ‘--hap-r2’ were used for values of allele frequency- and phased haplotype-based LD, respectively.

### Genomic population structure

Genomic population structure analysis was performed using principal component analysis (PCA). Because chromosomes 6 and 9 showed a higher number of variant sites than the other chromosomes, a PCA that directly used variant information as explanatory variables resulted in an overrepresentation of information on chromosomes 6 and 9. We performed a PCA as follows to avoid this problem. First, an additive relationship matrix based on function ‘A.mat’ in the R package *rrBLUP* version 4.6.2 (Endelman 2011) was constructed for each chromosome. Then an integrated additive relationship matrix was constructed using the following equation:


A=∑cAclc (1)

where A is the integrated additive relationship matrix, $A_{c}$ is the additive relationship matrix of chromosome *c*, and $l_{c}$ is the length of chromosome *c*. Finally, PCA with the function ‘prcomp’ in R version 4.3.1 (R Core Team 2023) was conducted for the integrated additive relationship matrix A.

### Genomic prediction

Phenotypic data for 96 of the 150 tomato cultivars used in this study were available from previous studies (Yamamoto *et al.* 2016, 2017). Because the phenotypic data were obtained from different years and cropping seasons, the mean phenotypic value of each cultivar was estimated by fitting a linear mixed model (LMM). The cultivar effect was treated as a fixed effect, whereas season and season × year were treated as random effects in the LMM. The LMM was fitted using function ‘lmer’ in the R package *lme4* version 1.1-34. We performed genomic predictions as follows to estimate the agronomic performance of the unphenotyped cultivars:


y=a+ε (2)

where y and ε are a vector of the cultivar effect obtained from the LMM and residuals, respectively. a is a vector of additive random effects as follows:


$$a=MVN(0,A\sigma_{A}^{2})$$ (3)

where *MVN* is the multivariate normal distribution and $\sigma_{A}^{2}$ is the variance for a. The genomic prediction model that accounts for additive-plus-dominance effect is as follows:


y=a+d+ε (4)

where d is a vector of dominance random effect with


$$d=MVN\left(0,D\sigma_{D}^{2}\right).$$ (5)

$\sigma_{D}^{2}$ is the variance for d. D is the integrated dominance relationship matrix calculated as


D=∑cDclc (6)

where $D_{c}$ is the dominance relationship matrix of chromosome *c*, calculated using function ‘D.mat’ in the R package *sommer* version 4.3.2 (Nishio and Satoh 2014). The genomic prediction was conducted using function ‘BGLR’ in the R package *BGLR* version 1.1.0 (Pérez and de Los Campos 2014) with arguments of nIter = 15000 and burnIn = 5000. Two-fold cross-validation was performed to evaluate the accuracy of the genomic prediction models used in this study. We performed 100 replicates for each trait, and the same fold was used for combinations of statistical models and traits. The predictive accuracy was measured as the Pearson’s correlation coefficient between the predicted and observed phenotypic values using the R function ‘cor.test’.

### GWAS

The GWAS was performed using a LMM as follows:


y=Sτ+Xβ+a+ε (7)

where S is a matrix for genotype values coded as {0, 1, 2} = {aa, Aa, AA}; τ is an additive marker effect; X is a matrix for fixed effect covariates (scores of principal components 1 and 2 in the population structure analysis in this study); and β is a coefficient vector for X. The solutions and statistical values of the LMMs were obtained using functions ‘lmm.diago’ and ‘lmm.diago.profile.likelihood’ in the R package *gaston* version 1.5.9, respectively (Perdry and Dandine-Roulland 2018). The statistical significance of the marker effect was evaluated using the log-likelihood ratio test, and the *P*-value of each test was calculated using the chi-square test. The false discovery rate of 0.05 was calculated using a method developed by Storey and Tibshirani (2003).

### Selection signatures

The detection of putative selection signatures in the breeding of Japanese large-sized fresh-market tomatoes was performed based on the composite likelihood ratio (CLR) test using SWEED version 3.2.1, with default parameter settings (Pavlidis *et al.* 2013). In addition, we compared the nucleotide diversity between all 150 cultivars and the cultivars released after 1990, where high sugar content became a primary breeding objective, to detect selection signatures contributing to recent tomato breeding in Japan. Selective sweeps in the cultivars released after 1990 were detected based on a reduction in nucleotide diversity (ROD). The nucleotide diversity π in the 150 cultivars (π_All_) and cultivars released after 1990 (π_1990_) were calculated using TASSEL version 5.0 with options window size 500 and step size 100 (Bradbury *et al.* 2007). Then ROD was calculated with π_All_/π_1990_. Since the cultivars used in this study included 147 F_1_, another possible selection signature was an unusual increase in heterozygosity. Genomic regions with unusual heterozygosity were detected using the difference between expected and observed heterozygosity based on the Hardy–Weinberg equilibrium (Huang *et al.* 2015). The difference between expected and observed heterozygosity (*H*) was calculated for the same windows used in the ROD calculation. Then *H*_All_ – *H*_1990_ was calculated as an indicator of unusual increases in heterozygosity.

### Data availability

The sequence data were deposited in the DNA Data Bank of the Japan Sequence Read Archive (DRA) under accession DRA008357.

## Results

### Genetic variation in the Japanese large-sized fresh-market tomato cultivars

**Fig. 1.**
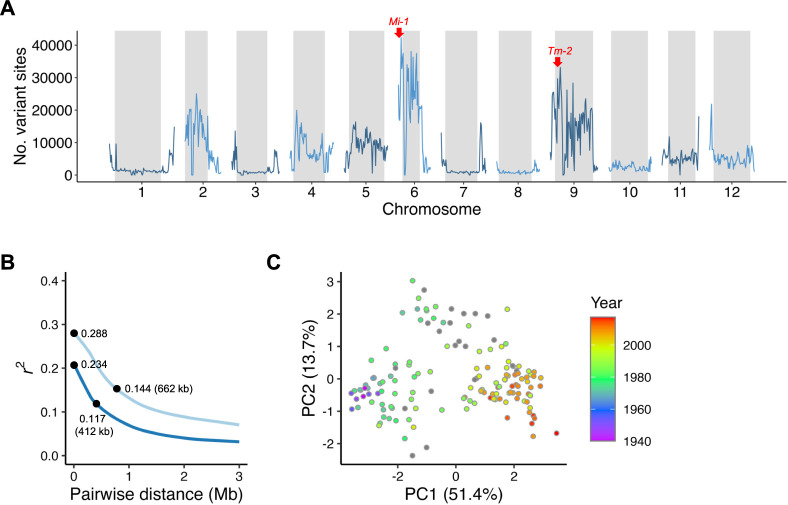
Whole-genome resequencing of the 150 Japanese tomato cultivars. (A) Genomic distribution of nucleotide variant sites detected in this study. The number of variant sites was counted within every 100-kb block along the genome sequence. The gray-shaded areas represent the centromeric regions. (B) Genome-wide average linkage disequilibrium (LD) decay in the 150 Japanese tomato cultivars. The light and dark blue lines indicate allele frequency-based and phased haplotype-based *r*^2^, respectively. (C) Population structure of the 150 Japanese tomato cultivars indicated using a principal component analysis. The dots indicate cultivars. The color of each dot indicates the release year of each cultivar.

Whole-genome resequencing of the 150 Japanese large-sized fresh-market tomato cultivars was conducted using short-read sequencing. The average read depth was 23–63× with the average equal to 41×, which resulted in the percentage of bases equal to or more than 5× coverage was 92–99% (Supplemental Table 1). After variant filtering (see Methods), 4,963,204 variant sites remained and were used for further analysis. Genome-wide distribution of the variant sites indicated that genomic regions, including resistance genes *Mi-1* (on chromosome 6) and *Tm-2* (on chromosome 9), showed a higher number of variant sites than other genomic regions (Fig. 1A). These results were reasonable because these regions have experienced introgression from wild species (Lin *et al.* 2014).

Decay of linkage disequilibrium (LD) was calculated to estimate the degree of intrachromosomal genome shuffling during Japanese tomato breeding. LD values were calculated based on allele frequency (i.e., genotype) and phased haplotypes. The latter was performed because most tomato cultivars in this study were F_1_, and allele frequency-based LD estimation may be disturbed by a high frequency of heterozygous genotypes. The *r*^2^ value decreased to half of its maximum value at approximately 662 kb (*r*^2^ = 0.144) and 412 kb (*r*^2^ = 0.117) in the allele frequency-based and phased haplotype-based method, respectively (Fig. 1B).

A PCA was performed to investigate the genomic population structure of the Japanese tomato cultivars (Fig. 1C). There was a high correlation between principal component (PC) 1 and the release year of each cultivar. In addition, cultivars released after 1990 were positioned in an area of high PC1 and low PC2 values.

### Changes in agronomic performance

We compared the agronomic performance and release year of 150 tomato cultivars to investigate the changes in the agronomic performance of Japanese large-sized fresh-market tomato cultivars over the past 80 years. The agronomic performance of the cultivar was estimated using data from two cropping seasons (Supplemental Table 2, Yamamoto *et al.* 2016, 2017). To estimate the agronomic performance of cultivars without phenotypic data, we performed genomic prediction and used the predicted values (i.e., genomic estimated breeding values [GEBVs]) as the agronomic performance of the cultivars. The additive-plus-dominance model showed little or no advantage over the additive model (Table 1). Therefore, we used the GEBVs from the additive model for further analyses. The estimated accuracy of the genomic prediction (*r*^2^) ranged from 0.199 to 0.693 (Table 1).

**Fig. 2.**
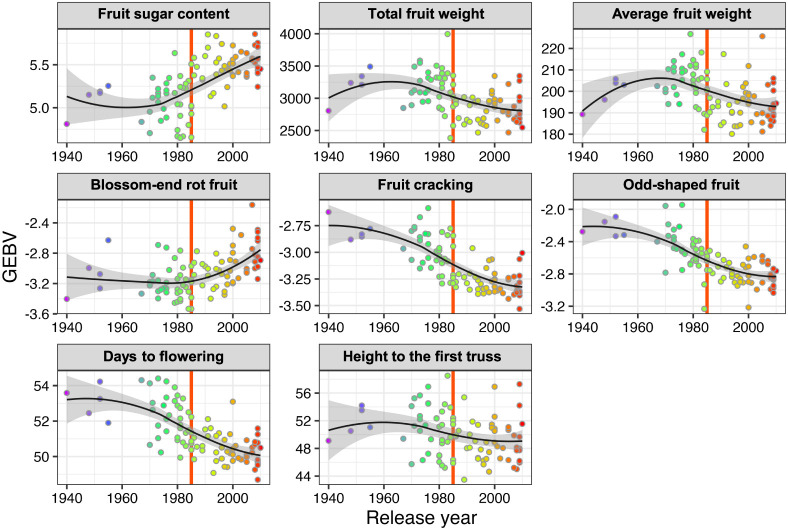
Agronomic performance of the 150 Japanese tomato cultivars estimated using genomic prediction. The dots in each panel indicate cultivars. The color of each dot indicates the release year of each cultivar, as in Fig. 1C. *x* and *y*-axes indicate the release year and genomic estimated breeding values (GEBVs) of cultivars, respectively. The values in blossom-end rot, fruit cracking, and odd-shaped fruit are empirical logit values of incidence ratio. The vertical red lines in the panels indicate 1985.

In the 1980s, trends in Japanese tomato breeding changed from high yield to good taste and flavor (Sumita *et al.* 2008, Tomato breeding team, Takii & Company, Limited 2020). ‘Momotaro’ (released in 1985) is a symbolic cultivar that contributed to this breeding trend change (Higashide *et al.* 2012). Our genomic predictions indicated that the fruit sugar content increased, whereas the yield (total fruit weight in this study) decreased after 1985 (Fig. 2). The average fruit weight showed a trend similar to the changes in total fruit weight. In addition, we observed selection for earlier days to flowering over the past 80 years (Fig. 2). The incidence of fruit physiological disorders (i.e., blossom-end rot fruit, fruit cracking, and odd-shaped fruit) is also an agronomically important trait. Our genomic predictions indicated that breeding selection over the past 80 years has decreased the incidence of fruit cracking and odd-shaped fruit (Fig. 2). However, the incidence of fruit blossom-end rot has increased in recently released cultivars (Fig. 2).

### Genetic loci associated with agronomic traits

We performed a GWAS for various agronomic traits to detect the genetic loci involved in the phenotypic variation in Japanese large-sized fresh-market tomato cultivars. Disease resistance is an important objective in tomato breeding. The highest signal for each disease resistance was detected close to previously identified genes/loci (Table 2, Supplemental Fig. 1).

**Fig. 3.**
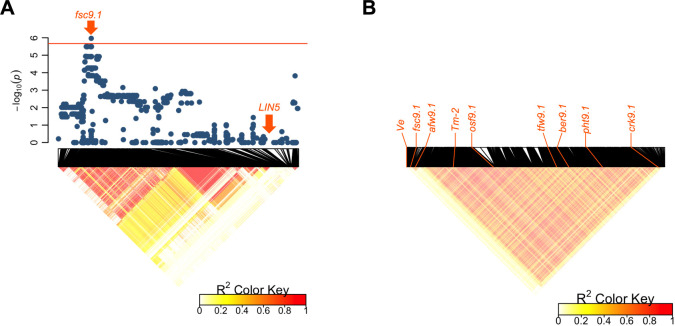
Genome-wide association study using the 150 Japanese tomato cultivars. (A) Local Manhattan plot (top) and LD heatmap (bottom) surrounding the significant signal for fruit sugar content on chromosome 9. The horizontal red line represents the threshold obtained from the 5% false discovery rate. (B) LD heatmap for chromosome 9. The red lines indicate the positions of genes or significant signals detected in this study.

We conducted a GWAS for quantitative traits (Table 2, Supplemental Fig. 2). The position of the significant signal for fruit sugar content (*fsc9.1*) was close to that of *LIN5*, a fruit-specific cell wall invertase gene involved in fruit sugar content (Fridman *et al.* 2004). However, whole-genome sequence-level analysis revealed that *fsc9.1* differs from *LIN5* because these loci were in different LD blocks (Fig. 3A). Moreover, no variant site was detected on ±5 kb region of *LIN5* and other cell wall invertase genes (Fridman and Zamir 2003). Significant signals for various traits were detected on chromosome 9 in the GWAS (Table 1). An LD heatmap for the entire region of chromosome 9 indicated that *Tm-2* (tomato mosaic virus [TMV] resistance locus), *osf9.1*, *tfw9.1*, *ber9.1*, and *phf9.1* are located on a large LD block that included the centromeric region (Table 2, Fig. 3B). To the best of our knowledge, no functionally characterized genes co-localized with the significant signals detected for quantitative traits in this study (Table 2).

### Selection signatures on the genome of the Japanese tomato cultivars

**Fig. 4.**
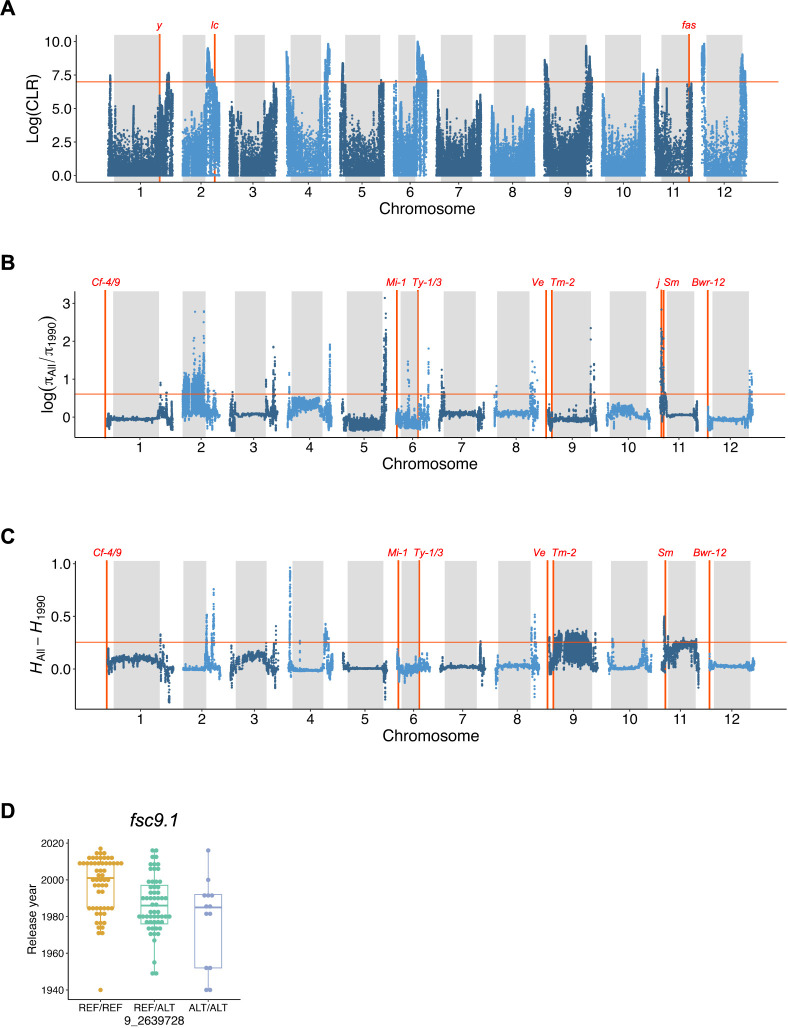
Selection signatures on the genomes of the 150 Japanese tomato cultivars. (A) Composite likelihood ratio (CLR) for the 150 Japanese tomato cultivars. (B) Nucleotide diversity reduction in the cultivars released after 1990 (π_1990_). It should be noted that *j* was not attributable to selection signatures on chromosome 11. (C) Unusual increase of heterozygosity in the cultivars released after 1990 (*H*_1990_). (A–C) The red horizontal line in each panel is the 95^th^ percentile of the values. (D) Boxplot for release year and *fsc9.1* genotype state of the tomato cultivars in this study.

We performed selective sweep detection using a likelihood-based method to explore the genomic regions of selection targets for the breeding of Japanese large-sized fresh-market tomatoes, (Fig. 4A). In the late 19^th^ century, when vegetable tomatoes were introduced to Japan, pink-colored fruits with many locules were preferred by Japanese people in those days (Sumita *et al.* 2008). Therefore, the genes related to these traits can be used as landmarks for selection signature detection. *y* is responsible for the pink fruit color (Ballester *et al.* 2010). In the Japanese tomato cultivars in this study, the 603-bp deletion on the promoter region and/or SNP on the 5ʹ splice site of the second intron was the functional nucleotide polymorphisms (FNPs) responsible for pink-colored fruit (Supplemental Table 3, Veerappan *et al.* 2016). The composite likelihood ratio (CLR) of the genomic region including the FNPs was approximately 10% from the top (Fig. 4A). The major loci that control locule number in tomatoes are *lc* and *fas* (Cong *et al.* 2008, Munos *et al.* 2011). The two putative FNPs in the *lc* promoter region were alleles that increased the locule number in the 150 cultivars used in this study (Supplemental Table 3). The CLR of the genomic region including these FNPs was approximately 5% from the top (Fig. 4A). In contrast, the CLR of the genomic region including *fas* was approximately 20% from the top, indicating that *fas* was not a target of selection for high locule numbers in the Japanese tomato cultivars (Fig. 4A). Although *y* and *lc* are important loci that characterize the features of Japanese large-sized fresh-market tomatoes, many other genomic regions with putative selection signatures showed higher CLR values (Fig. 4A).

High fruit sugar content became a major breeding objective in the 1980s (Higashide *et al.* 2012). Consistent with this breeding objective change, our genomic prediction indicated changes in agronomic performance, not only in fruit sugar content but also in other traits (Fig. 2). We compared the genomic diversity of all 150 cultivars and cultivars released after 1990 to detect the selection signatures for this breeding objective change and the subsequent agronomic performance changes (Fig. 4B, 4C). First, the ROD was calculated to detect putative selective sweeps. The ROD value of the genomic region including *j*, a gene involved in jointless pedicles (Mao *et al.* 2000), was 0.6% from the top, suggesting that this region was a selection target in the cultivars released after 1990 (Fig. 4B). However, there were no allelic variations in the FNPs of *j* indicating that the selective sweep was not attributable to the selection of *j* itself (Supplemental Table 3). There was another putative selection signature close to the genomic region including *j*, which was 5% from the top (Fig. 4B). The putative selective sweep was close to *Sm*, a gene that confers resistance to gray leaf spots (Table 2). An unusual increase in heterozygosity in the cultivars released after 1990 was observed in the centromeric region of chromosome 9, where *Tm-2* was located (Fig. 4C). It has been reported that introgression of the resistance allele of *Tm-2* from the wild species (*S. peruvianum*) is accompanied by a linkage drag of ~60 Mb of the genomic region that includes the entire centromeric region (van Rengs *et al.* 2022). Therefore, the unusual increase in heterozygosity in the centromeric region of chromosome 9 was attributed to selection for *Tm-2*.

Notably, despite conferring disease resistance being an important objective in tomato breeding, resistance genes, except for *Sm* and *Tm-2*, were not co-localized with putative selection signatures (Table 2, Fig. 4B, 4C). In addition, except for the QTLs on the centromeric region of chromosome 9 (unusual heterozygosity increase), the putative QTLs detected by the GWAS did not co-localize with any putative selection signature (Table 2, Fig. 4B, 4C). For example, although fruit sugar content is an important breeding target (Fig. 2), *fsc9.1* was not detected in our selection signature analyses (Fig. 4B, 4C). For *fsc9.1*, the recently released cultivars possessed a reference genome-type allele even though the reference genome-type allele showed a decrease in fruit sugar content (Fig. 4D). These results indicated that *fsc9.1* is not a QTL that contributes to an increase in fruit sugar content in Japanese tomato breeding.

## Discussion

Intensive selection for good taste and flavor resulted in a decreased yield performance in Japanese tomato breeding (Higashide *et al.* 2012). Our genomic prediction analysis was in good agreement with these changes (Fig. 2). In addition, we found a selection for earlier days to flowering (Fig. 2). This selection seems reasonable because early flowering in tomatoes would result in a longer harvest period and a higher yield per cultivation season. Japanese tomato breeding decreased the incidence of fruit cracking and odd-shaped fruits (Fig. 2). However, the incidence of blossom-end rot increased, especially in cultivars released after the 1990s (Fig. 2). Although extensive research has demonstrated that calcium homeostasis and reactive oxygen species are important players in the incidence of blossom-end rot fruit, the mechanisms involved remain elusive (Topcu *et al.* 2022). A recent study found that a mutant resistance to blossom-end rot shows a decrease in sugar content in ripe fruit (Nicolas *et al.* 2023). This suggests that sugar metabolism plays an important role in blossom-end rot. Decreasing the incidence of blossom-end rot without decreasing the fruit sugar content is another important problem that needs to be solved in future tomato breeding.

The additive-plus-dominance model showed little or no advantage over the additive model in our genomic prediction analysis (Table 1). In general, large datasets are required to detect the advantages of dominance effects in genomic prediction (Varona *et al.* 2018). Therefore, based on our genomic prediction results, it is difficult to conclude how much the dominance effect contributes to Japanese tomato breeding. Another possibility for the insignificant advantage of the additive plus dominance model in our analysis is that many variant loci have only two genotypes, consisting of heterozygous and homozygous of an allele; therefore, estimation of the dominance effect was impossible for the loci. This is because the plant materials in this study were not random populations (i.e., elite cultivars). Using experimentally designed populations is necessary to estimate the contribution of dominance effect on Japanese tomato breeding (Torgeman and Zamir 2023).

The most significant signals for resistance genes in the GWAS were detected close to, but slightly different from the positions of the previously identified genes (Table 2, Supplemental Fig. 2). For example, the causal gene of *Tm-2* is located from 13,618,955 to 13,621,829 bp of chromosome 9 (Lanfermeijer *et al.* 2005), whereas the highest significant signal for TMV resistance in our GWAS was detected at 10,683,562 bp (Table 2). Two resistance alleles for the *Tm-2* locus have been used for tomato breeding (Lanfermeijer *et al.* 2005). Thus, the *Tm-2* locus in Japanese tomato cultivars contains multiple alleles. The analysis of phenotypic variation derived from multi-allelic genes using bi-allelic markers in GWAS often causes a positional gap between the highest significant signal and the true causal gene (Dickson *et al.* 2010). Haplotype-based GWAS is an effective method to avoid this problem (Yano *et al.* 2016). However, estimating the haplotype state of the *Tm-2* region using short-read sequencing is difficult because of complicated structural variations in the genomic region (van Rengs *et al.* 2022). Genotyping using long-read sequencing is necessary for GWAS to locate significant signals precisely in such a case (Alonge *et al.* 2020).

Fruits with high locule numbers are an important feature of Japanese large-sized fresh-market tomatoes (Sumita *et al.* 2008, Tomato breeding team, Takii & Company, Limited 2020). Whole-genome resequencing and selection signature analyses indicated that *lc* plays an important role in this feature (Fig. 4A, Supplemental Table 3). In contrast, *fas* (another well-known locus for high locule numbers) was not involved in the breeding of Japanese large-sized fresh-market tomatoes (Fig. 4A, Supplemental Table 3). This is probably because using the *fas* allele leads to an increase in odd-shaped fruits (Chu *et al.* 2019). The *Tm-2* genomic region was detected as a selection signature region that showed an unusual increase in heterozygosity after the 1990s (Fig. 4). The heterozygous state of *Tm-2*^a^ allele shows higher fruit yield than the homozygous state (Tanksley *et al.* 1998). Therefore, the unusual heterozygosity increase in *Tm-2* would be not only for resistance to TMV but also for higher yield performance. Actually, we detected a significant signal for total fruit weight (*tfw9.1*) in the LD block including *Tm-2* (Table 2, Fig. 3B). However, as indicated in a previous study, it is difficult to determine whether *tfw9.1* is a pleiotropic effect of *Tm-2*^a^ itself or gene(s) associated with linkage drag because of the low recombination frequency in the region (Tanksley *et al.* 1998). Although fruit sugar content and total fruit weight showed drastic changes from the 1980s to the 1990s (Fig. 2), the significant signals in our GWAS did not co-localize with the regions detected in our selection signature analysis (Fig. 4). One possible explanation for this inconsistency is the low detection power of our GWAS owing to the small number of samples. Another possibility is that this inconsistency was caused by collinearity between phenotypic values and population structure. Among the 150 cultivars, the genomic population structure (PC1 and PC2) was related to the release year (Fig. 1C). In addition, many agronomic traits analyzed in this study showed an increase or decrease with the release year of the cultivars (Fig. 2). Thus, there is collinearity between the genomic population structure and phenotypic values. Since the genomic population structure was incorporated as a covariate in a linear-mixed model of GWAS, it is possible that the QTL effect related to agronomic traits in Japanese tomato cultivars was negated by the covariates derived from the genomic population structure. However, GWAS using covariates from the genomic population structure is generally necessary to avoid false positives (Yu *et al.* 2006). Therefore, developing genetic mapping populations that were free from population structure problems is necessary to clarify the inconsistency between our GWAS and selection signature analyses (Ohyama *et al.* 2023).

In summary, our whole-genome resequencing of 150 Japanese large-sized fresh-market tomato cultivars revealed how breeding selection changed the genomes of Japanese cultivars and provided basic information for Japanese tomato breeding. Although our analyses focused elucidating historical tomato breeding in Japan, we expect that the nucleotide variant information and some putative QTLs detected in this study will contribute to future tomato breeding.

## Author Contribution Statement

E.Y. designed the study and wrote the manuscript; H.M., A.O., T.N., H.Y., and K.M. performed the phenotype analyses; K.S. and S.I. performed the sequence analyses.

## Supplementary Material

Supplemental Figures

Supplemental Tables

## Figures and Tables

**Table 1.** Accuracy of genomic prediction models

Trait	Model*^a^*	
	A	A + D
Fruit sugar content	0.693 ± 0.045	0.670 ± 0.045
Total fruit weight	0.496 ± 0.064	0.485 ± 0.064
Average fruit weight	0.199 ± 0.084	0.177 ± 0.084
Blossom-end rot fruit	0.452 ± 0.078	0.406 ± 0.085
Fruit cracking	0.227 ± 0.090	0.208 ± 0.090
Odd-shaped fruit	0.369 ± 0.063	0.310 ± 0.064
Days to flowering	0.628 ± 0.036	0.648 ± 0.036
Height to the first truss	0.507 ± 0.062	0.477 ± 0.073

Accuracy was evaluated as a Pearson’s correlation coefficient between phenotypic and predicted values from 100 cycles of Two-fold cross-validation. The values are mean ± standard deviation. *^a^* A and A + D indicate additive and additive-plus-dominance effect models, respectively.

**Table 2.** The significant associations detected in the GWAS

Category	Trait	Information on the highest signal					Locus name	Putative causal gene	Reference
		Chromosome	Position (bp)	–log_10_*P*	Effect_REF/REF_	Effect_ALT/ALT_			
Resistance	Root-knot nematode	SL3.0ch06	2264323	5.54	–	–	*Mi-1*	*Mi-1*	Kaloshian *et al.* (1998)
	Tobacco mosaic virus	SL3.0ch09	10683562	4.77	–	–	*Tm-2*	*Tm-2*	Tanksley *et al.* (1998)
	Leaf mold	SL3.0ch01	1120544	8.35	–	–	*Cf-4/9*	*Cf-4/9*	Dixon *et al.* (1996)
	Bacterial wilt	SL3.0ch12	3232956	7.18	–	–	*Bwr-12*	*Bwr-12*	Kim *et al.* (2018)
	Verticillium wilt	SL3.0ch09	331553	4.38	–	–	*Ve*	*Ve*	Kawchuk *et al.* (2001)
	Gray leaf spot	SL3.0ch11	6959989	4.01	–	–	*Sm*	*Sm*	Su *et al.* (2019)
	Tomato yellow leaf curl virus	SL3.0ch06	34381855	4.21	–	–	*Ty-1/3*	*Ty-1/3*	Zamir *et al.* (1994)
Quantitative	Fruit Sugar content (˚Brix)	SL3.0ch09	2639728	5.74	0.00	0.26	*fsc9.1*	–	–
	Total fruit weight (g)	SL3.0ch09	39514471	4.52	0.00	428.72	*tfw9.1*	–	–
		SL3.0ch12	2894309	4.48	0.00	461.34	*tfw12.1*	–	–
	Average fruit weight (g)	SL3.0ch09	5256841	6.39	0.00	33.02	*afw9.1*		–
	Blossom-end rot (%)	SL3.0ch09	42378005	5.98	12.00	9.10	*ber9.1*	–	–
	Fruit cracking (%)	SL3.0ch09	63033671	6.91	12.00	17.00	*fck9.1*	–	–
	Odd-shaped fruit (%)	SL3.0ch09	18327575	5.53	12.00	9.50	*osf9.1*	–	–
	Days to flowering	SL3.0ch02	26914589	4.63	0.00	1.59	*dtf2.1*	–	–
	Height to the first truss (cm)	SL3.0ch09	50246026	5.91	0.00	5.50	*phf9.1*	–	–
