# Supplementary material for: Whole-genome resequencing of Japanese large-sized tomato cultivars provides insights into the history of modern breeding
Source: Breed Sci. 2024 Aug 23;74(4):344–53. doi: 10.1270/jsbbs.24004 (PMC11769584; doi:10.1270/jsbbs.24004)
Supplement: Supplementary file 1 — Supplemental Figures [file 74_344_s1.pdf]

doi: 10.1270/jsbbs.24004

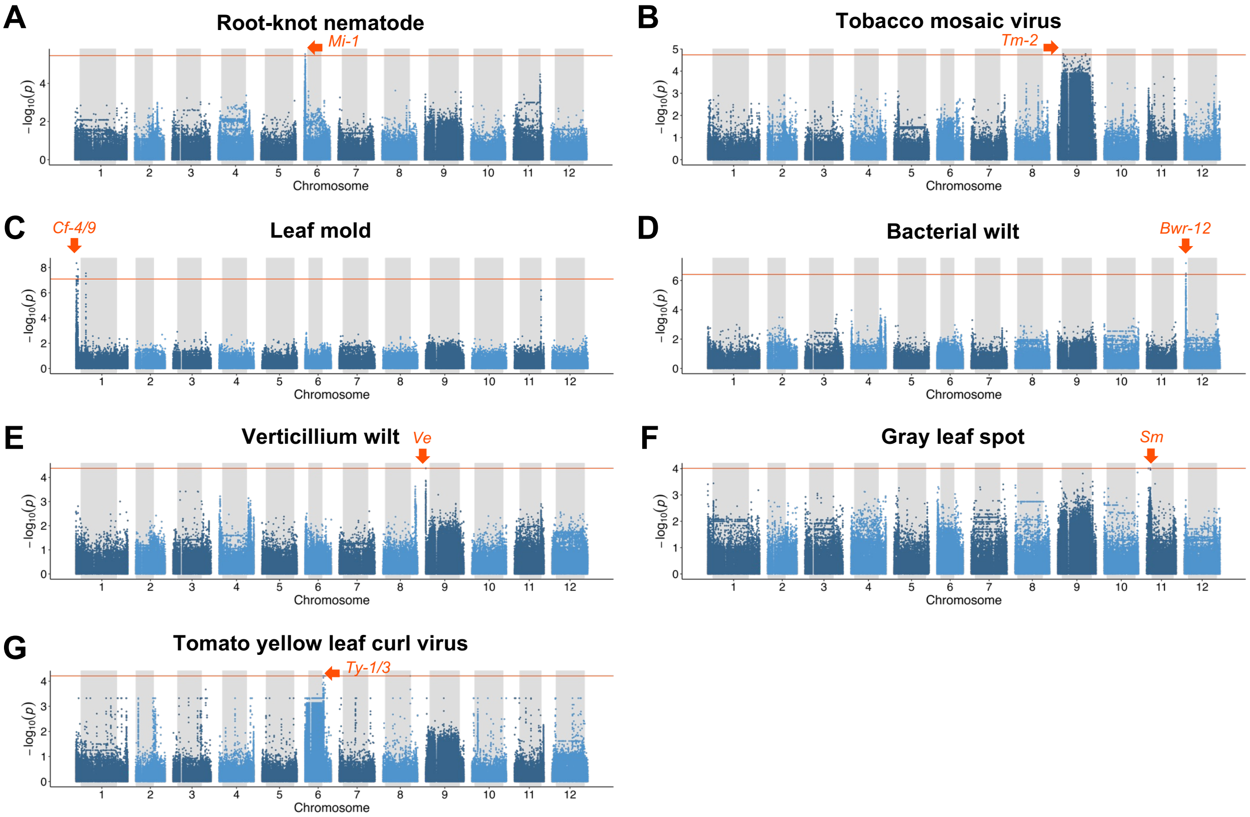

Supplemental Fig. 1. Manhattan plots of genome-wide association study for disease resistance. (A) Root-knot nematode. (B) Tobacco mosaic virus. (C) Leaf mold. (D) Bacterial wilt. (E) Verticillium wilt. (F) Gray leaf spot. (G) Tomato yellow leaf curl virus. (A)-(G). The red horizontal lines indicate false discovery rates of 5%.

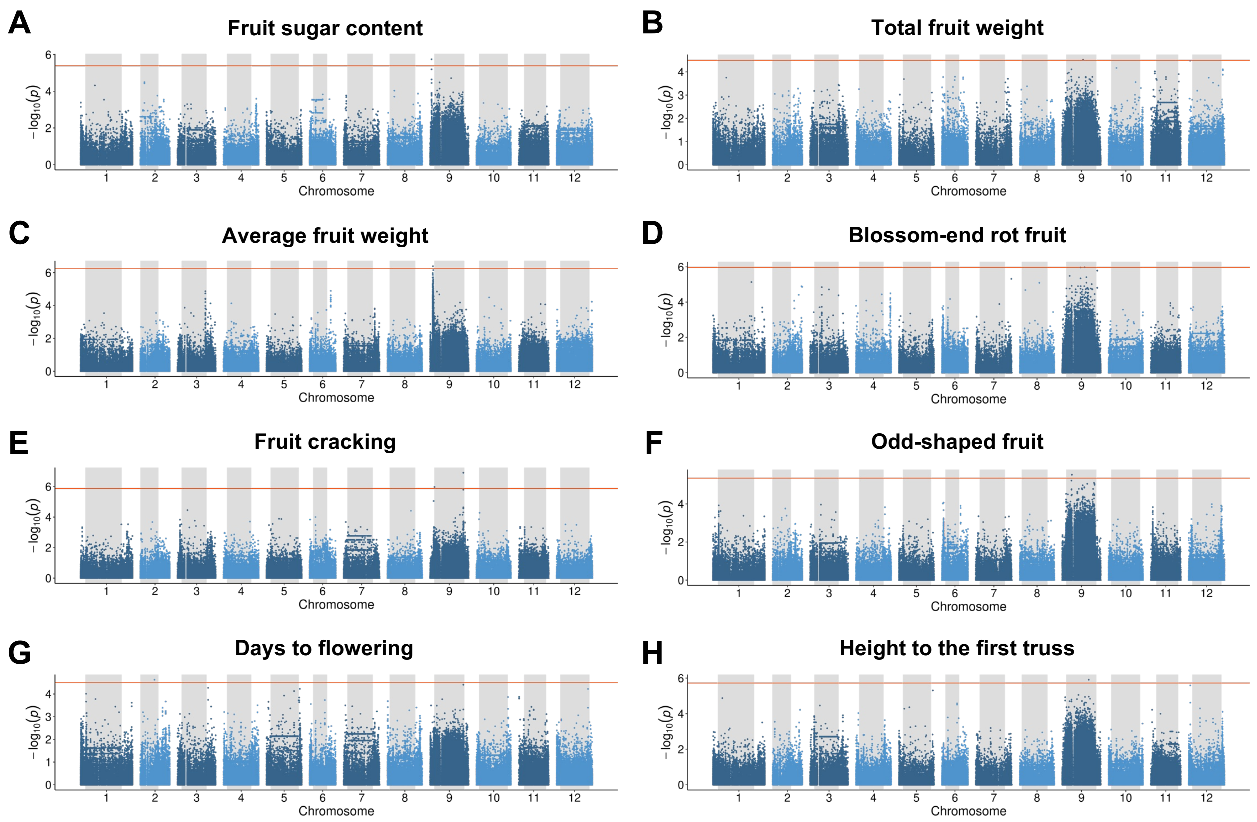

Supplemental Fig. 2. Manhattan plots of genome-wide association study for quantitative traits. (A) Fruit sugar content. (B) Total fruit weight. (C) Average fruit weight. (D) Blossom-end rot. (E) Fruit cracking. (F) Odd-shaped fruit. (G) Days to flowering. (H) Height to the first truss. (A)-(H). The red horizontal lines indicate false discovery rates of 5%.
