# Supplementary material for: Whole-genome resequencing of Japanese large-sized tomato cultivars provides insights into the history of modern breeding
Source: Breed Sci. 2024 Aug 23;74(4):344–53. doi: 10.1270/jsbbs.24004 (PMC11769584; doi:10.1270/jsbbs.24004)
Supplement: Supplementary file 2 — Supplemental Tables [file 74_344_s2.pdf]

Supplemental Table 1. Whole-genome resequencing of the 150 Japanese tomato cultivars.

Cultivar	Type	Release year	Total reads	Map rate (%)	Coverage (%)			Depth
					1x	5x	10x	
SL1	F_1	2009	202,496,203	95.93	99.85	99.62	98.70	35.785
SL2	F_1	1985	214,841,237	95.93	99.68	99.44	98.66	37.850
SL3	F_1	1998	193,793,288	95.77	99.81	99.50	98.28	34.137
SL4	F_1	1975	189,162,531	96.64	99.81	99.50	98.41	34.036
SL5	F_1	1985	262,253,679	95.90	99.74	99.57	99.12	46.276
SL6	F_1	2005	204,546,957	95.44	99.76	99.42	98.33	36.251
SL7	F_1	NA	195,280,213	95.72	99.65	99.28	97.78	34.302
SL8	F_1	1971	227,272,847	97.06	99.81	99.60	99.01	41.214
SL9	F_1	2000	206,702,423	95.97	99.81	99.52	98.45	36.578
SL10	F_1	1955	248,474,556	96.99	99.82	99.65	99.21	44.374
SL11	F_1	1983	208,873,594	96.05	99.69	99.43	98.49	36.987
SL12	F_1	1990	220,186,910	95.91	99.81	99.57	98.75	38.934
SL13	F_1	1995	211,107,279	95.98	99.81	99.55	98.63	37.299
SL14	F_1	1991	222,704,269	96.07	99.82	99.59	98.84	39.390
SL15	F_1	1976	225,406,881	96.32	99.72	99.51	98.84	39.933
SL16	F_1	2003	217,903,386	95.91	99.82	99.58	98.77	38.578
SL17	F_1	1976	242,379,363	95.44	99.82	99.63	99.02	42.488
SL18	F_1	2000	218,560,770	96.63	99.80	99.57	98.98	39.042
SL20	F_1	1976	239,260,298	95.93	99.72	99.51	98.88	42.364
SL21	F_1	1952	226,282,956	96.77	99.70	99.51	99.02	40.453
SL22	F_1	1991	180,628,782	95.85	99.80	99.44	97.86	31.904
SL23	F_1	1996	181,341,988	95.81	99.80	99.45	97.98	32.093
SL24	F_1	1987	271,204,001	95.93	99.82	99.65	99.22	47.977
SL25	F_1	1979	181,847,804	95.93	99.70	99.36	97.90	32.238
SL26	F_1	2005	212,672,642	95.51	99.84	99.58	98.52	37.273
SL27	F_1	1994	202,588,717	96.74	99.85	99.62	98.87	36.196
SL28	F_1	1980	243,773,705	96.67	99.80	99.59	99.14	43.443
SL29	F_1	1979	228,225,761	96.30	99.68	99.42	98.66	40.767
SL30	F_1	2008	242,128,585	95.44	99.80	99.58	98.90	42.540
SL31	F_1	NA	224,729,489	96.34	99.53	99.23	98.50	39.654
SL32	F_1	2007	238,470,393	95.74	99.84	99.64	99.03	41.959
SL33	F_1	1967	222,566,493	96.92	99.85	99.68	99.18	39.875
SL34	F_1	2009	195,052,559	95.93	99.80	99.50	98.34	34.476
SL35	F_1	1977	187,046,473	95.68	99.68	99.33	97.75	32.923
SL36	F_1	1980	190,589,213	96.83	99.86	99.62	98.70	34.092
SL37	F_1	1973	184,035,444	95.84	99.69	99.36	97.87	32.530
SL38	F_1	1989	245,512,771	96.86	99.81	99.63	99.21	43.919
SL39	F_1	2001	232,266,017	95.97	99.85	99.61	98.99	41.104
SL40	F_1	1992	209,862,634	95.60	99.73	99.48	98.58	36.965
SL41	F_1	1996	193,090,048	95.49	99.79	99.45	98.02	33.854
SL42	F_1	1997	270,483,098	95.32	99.77	99.55	99.00	47.484
SL43	F_1	1985	196,916,597	94.88	99.69	99.34	97.88	34.383
SL44	F_1	1982	257,206,669	95.92	99.67	99.43	98.85	45.826
SL45	F_1	2002	287,823,569	95.50	99.63	99.40	98.82	50.664
SL46	F_1	1970	186,834,145	96.49	99.76	99.46	98.32	33.112
SL47	F_1	NA	264,835,683	88.15	99.80	99.60	99.01	42.771
SL48	F_1	1999	268,086,390	95.56	99.66	99.38	98.66	47.167
SL49	F_1	1989	297,648,084	95.24	99.88	99.75	99.36	51.525
SL50	F_1	1970	236,783,143	96.79	99.65	99.46	98.98	42.134
SL51	F_1	1971	193,064,278	96.32	99.86	99.58	98.31	33.920
SL52	F_1	1998	209,025,921	96.30	99.63	99.32	98.36	37.264
SL53	F_1	1981	224,272,383	95.35	99.72	99.48	98.63	38.950
SL54	F_1	2009	222,238,909	95.51	99.81	99.56	98.66	38.667

Supplemental Table 1. *Continued*.

Cultivar	Type	Release year	Total reads	Map rate (%)	Coverage (%)			Depth
					1x	5x	10x	
SL55	F_1	1980	251,345,719	96.31	99.86	99.71	99.29	44.176
SL56	F_1	1976	144,966,717	97.34	99.80	99.29	96.83	26.490
SL57	F_1	1984	230,657,347	95.05	98.02	97.10	96.18	40.173
SL58	Pure-line	Before 1940	240,691,551	97.44	99.61	99.40	98.92	44.091
SL59	F_1	2010	203,837,322	95.14	99.63	99.24	97.84	36.003
SL60	F_1	NA	244,972,791	96.12	99.88	99.72	99.21	43.300
SL61	F_1	2009	253,694,783	95.74	99.78	99.52	98.80	44.982
SL62	F_1	1952	212,174,364	96.89	99.68	99.44	98.65	37.536
SL63	F_1	1980	222,655,666	96.89	99.71	99.52	98.96	39.761
SL64	F_1	1984	202,594,248	96.25	99.83	99.53	98.40	36.381
SL65	F_1	1983	247,369,066	97.31	99.67	99.49	99.05	45.276
SL66	F_1	2005	155,775,781	96.23	99.73	99.18	96.69	28.118
SL67	F_1	1993	261,459,954	96.12	99.65	99.43	98.88	47.105
SL68	F_1	2008	231,420,840	96.23	99.78	99.52	98.75	41.766
SL69	F_1	1973	248,957,861	96.65	99.79	99.58	98.97	44.978
SL70	F_1	2000	236,451,503	95.66	99.71	99.44	98.57	41.877
SL71	F_1	1981	178,671,060	96.00	99.65	99.25	97.67	32.063
SL72	F_1	2005	221,122,431	96.15	99.77	99.50	98.68	39.839
SL73	F_1	NA	224,136,408	96.30	99.70	99.45	98.73	40.535
SL74	F_1	2009	259,815,844	95.99	99.80	99.59	99.04	46.584
SL75	F_1	2009	223,808,358	95.97	99.75	99.46	98.53	40.151
SL77	F_1	1982	265,330,487	97.01	99.84	99.66	99.21	48.183
SL79	F_1	1985	258,926,142	97.30	99.77	99.57	99.13	47.348
SL80	F_1	1994	239,670,722	96.05	99.75	99.47	98.69	43.056
SL81	F_1	1974	201,961,228	97.18	99.81	99.53	98.72	36.797
SL82	F_1	1981	220,475,175	95.62	99.74	99.45	98.54	39.332
SL83	F_1	1986	247,703,386	95.64	99.80	99.56	98.87	44.306
SL84	F_1	2007	205,261,787	96.02	99.77	99.44	98.33	36.888
SL85	F_1	1994	251,960,842	96.30	99.77	99.54	98.95	45.118
SL86	F_1	1999	227,572,368	96.20	99.68	99.40	98.55	40.897
SL87	F_1	2009	283,372,642	94.31	97.91	97.01	96.14	49.429
SL88	F_1	2000	221,455,443	95.96	99.68	99.37	98.33	39.722
SL89	F_1	1981	274,057,955	95.89	99.83	99.62	98.98	49.041
SL90	F_1	2008	260,811,996	96.28	99.78	99.56	98.99	47.033
SL91	F_1	1986	222,165,010	95.55	99.79	99.48	98.40	39.616
SL92	F_1	2000	213,561,918	94.75	97.72	96.63	95.01	37.559
SL93	F_1	2009	203,003,195	94.51	97.81	96.75	95.30	35.834
SL94	F_1	1997	299,526,852	95.35	99.72	99.54	99.09	51.360
SL95	F_1	1985	280,582,990	95.91	99.79	99.61	99.18	48.625
SL96	F_1	1981	301,255,660	96.26	99.58	99.41	99.08	52.197
SL101	F_1	1973	305,767,462	96.05	99.84	99.70	99.37	54.784
SL102	F_1	1973	240,869,979	97.65	99.68	99.49	99.06	44.334
SL103	F_1	1948	290,135,250	97.20	99.83	99.69	99.36	52.873
A-001	F_1	2016	278,680,325	94.16	94.27	92.85	91.42	33.899
A-002	F_1	2016	315,562,882	94.16	94.55	93.29	92.10	40.423
PS-001	F_1	NA	237,705,606	96.21	99.63	99.38	98.67	41.971
PS-002	F_1	NA	206,572,451	96.12	99.85	99.56	98.49	36.752
PS-003	F_1	NA	237,530,886	97.40	99.83	99.65	99.15	43.048
PS-007	F_1	2014	227,022,672	94.43	97.92	96.94	95.81	39.603
PS-008	F_1	2017	277,519,509	94.42	97.95	97.02	96.14	46.902
PS-009	F_1	NA	206,769,539	97.22	99.79	99.52	98.73	37.200
PS-012	F_1	1996	263,938,553	97.34	99.81	99.63	99.24	47.780
PS-016	F_1	1992	223,254,159	97.71	99.77	99.53	98.96	40.732

Supplemental Table 1. *Continued*.

Cultivar	Type	Release year	Total reads	Map rate (%)	Coverage (%)			Depth
					1x	5x	10x	
PS-020	F_1	1977	208,936,824	97.55	99.68	99.44	98.71	37.909
PS-021	F_1	NA	212,934,623	95.59	97.79	96.81	95.81	37.698
PS-022	F_1	NA	212,009,456	95.57	99.73	99.47	98.50	37.527
PS-023	F_1	NA	234,835,318	97.61	99.69	99.49	99.00	42.738
PS-024	F_1	NA	228,787,186	95.49	94.75	93.35	91.79	29.951
PS-025	F_1	1980	291,290,732	97.67	99.86	99.74	99.46	53.057
PS-026	F_1	1978	188,913,915	95.43	98.13	97.08	95.64	33.421
PS-029	F_1	NA	209,434,507	97.25	99.77	99.51	98.79	37.848
PS-030	F_1	1972	213,612,188	97.45	99.67	99.42	98.77	38.497
PS-033	F_1	2012	209,567,349	96.50	99.76	99.47	98.59	37.582
PS-038	F_1	2010	293,372,313	96.45	99.78	99.60	99.23	52.570
PS-042	Pure-line	Before 1940	145,491,348	95.13	98.61	98.06	97.2	39.143
PS-043	F_1	1982	192,936,202	95.86	98.57	97.39	93.73	23.836
PS-044	F_1	NA	244,610,054	96.31	99.82	99.61	98.98	43.288
PS-046	F_1	2011	204,205,485	95.51	99.80	99.49	98.31	36.176
PS-047	F_1	2011	220,720,425	95.48	99.80	99.53	98.56	39.032
PS-048	F_1	1990	256,319,359	97.40	99.80	99.62	99.21	44.173
PS-051	F_1	NA	280,078,349	97.58	99.84	99.71	99.39	50.909
PS-053	F_1	1984	208,174,545	97.53	99.75	99.50	98.79	37.816
PS-054	F_1	NA	210,283,800	97.48	99.62	99.35	98.67	38.175
PS-056	F_1	1976	271,414,172	96.53	99.68	99.50	99.05	48.514
PS-058	F_1	1989	198,113,783	96.57	99.81	99.51	98.47	35.577
PS-059	F_1	2012	135,773,065	96.15	99.38	98.51	94.15	24.169
PS-060	F_1	2012	233,717,117	96.19	99.83	99.58	98.82	41.459
PS-061	F_1	2015	339,907,374	95.13	99.68	99.51	99.16	59.252
PS-062	F_1	2013	199,958,472	94.48	97.78	96.68	95.21	34.821
PS-063	F_1	1950	226,754,089	97.49	99.68	99.47	98.92	41.070
PS-064	F_1	1989	242,933,859	97.74	99.86	99.70	99.27	44.250
PS-066	F_1	2013	228,511,115	95.29	99.80	99.52	98.58	40.137
PS-067	F_1	NA	173,930,557	95.06	99.78	99.28	96.92	30.581
PS-069	F_1	NA	263,063,576	97.54	99.83	99.67	99.27	47.679
PS-070	Pure-line	Before 1940	251,276,791	97.41	99.50	99.29	98.86	45.338
PS-072	F_1	1998	207,581,885	97.50	99.63	99.36	98.68	37.700
PS-074	F_1	NA	239,275,039	96.71	99.79	99.57	98.96	42.932
PS-075	F_1	NA	199,451,766	97.60	99.83	99.59	98.85	36.211
PS-076	F_1	NA	263,541,962	97.57	99.83	99.68	99.29	47.732
PS-079	F_1	2011	205,530,554	96.45	99.77	99.48	98.50	36.743
PS-082	F_1	2008	229,431,398	96.26	99.80	99.57	98.85	40.844
PS-084	F_1	2009	207,422,104	96.33	99.78	99.49	98.50	36.985
PS-087	F_1	NA	269,771,505	96.32	99.80	99.61	99.12	48.040
PS-089	F_1	2012	254,178,452	96.47	99.79	99.58	99.03	45.375
PS-093	F_1	2016	178,118,085	96.09	99.66	99.20	97.16	31.317
PS-094	F_1	2015	367,707,595	95.34	99.56	99.34	99.01	63.333
PS-096	F_1	1992	267,548,417	96.20	99.70	99.51	98.95	47.063

Supplemental Table 2. Estimated ratio of variance components in linear mixed modeling.

Trait	Cultivar	Season × year	Season	No. of fruits	Residual
Fruit sugar content	0.241	0.061	0.470	n.a.	0.229
Total fruit weight	0.181	0.559	0.000	n.a.	0.260
Average fruit weight	0.246	0.384	0.001	n.a.	0.368
Blossom-end rot	0.179	0.278	0.001	0.002	0.542
Fruit cracking	0.582	n.a.	n.a.	0.000	0.418
Odd-shaped fruit	0.275	0.033	0.027	0.014	0.647
Days to flowering	0.017	0.284	0.662	n.a.	0.037
Height to the first truss	0.036	0.124	0.755	n.a.	0.085

n.a.: Not analyzed because of no variation in the factor in the trait.

Supplemental Table 3. Allelic distribution of functional nucleotide polymorphisms (FNPs) in the 150 Japanese tomato cultivars.

Locus	Chromosome	FNP Position (bp)	REF	ALT	No. of cultivars			Note
					REF-homo	Hetero	ALT-homo	
y	SL3.0ch01	600,223-78,599,620	Ins	Del	10	0	140	ALT-homo causes pink-colored fruit
		78,605,440	G	T	145	5	0	ALT-homo causes pink-colored fruit
lc	SL3.0ch02	47,743,766	T	C	0	0	150	ALT-homo causes fruit with high locule numbers
		47,743,772	A	G	0	0	150	ALT-homo causes fruit with high locule numbers
